# Psychometrics of the Persian version of the program in palliative care education and practice questionnaire (German revised - PPCEP-GR)

**DOI:** 10.1186/s12904-023-01196-3

**Published:** 2023-06-23

**Authors:** Mohajer Abdoli, Katharina Fetz, Shahram Molavynejad, Hamid Sharif-Nia, Marziyeh Asadizaker

**Affiliations:** 1 Nursing Care Research Center in Chronic Diseases, School of Nursing & Midwifery, Ahvaz Jundishapur University of Medical Sciences, Ahvaz, Iran; 2 Chair of Research Methodology and Statistics, Department of Psychology and Psychotherapy, Faculty of Health, Witten/Herdecke University, Witten, Germany; 3 Institute for Research in Operative Medicine, Department Biometrics and Registry Data Research, Witten/Herdecke University, Witten, Germany; 4 Department of Anaesthesiology and Operative Intensive Care, Cologne-Merheim Medical Centre, Cologne, Germany; 5 Institute for Emergency Medicine, University Hospital Schleswig-Holstein, Kiel, Germany; 6 Traditional and Complementary Medicine Research Center, Addiction Institute, Mazandaran University of Medical Sciences, Sari, Iran; 7 Department of Nursing, Amol Faculty of Nursing and Midwifery, Mazandaran University of Medical Sciences, Sari, Iran

**Keywords:** Program in Palliative Care Education and Practice Questionnaire, Psychometric, Validity, Reliability

## Abstract

**Background:**

In order to improve the provision of palliative care by nurses, it is necessary to have a tool that measures different dimensions of palliative care and the knowledge and performance of nurses in this field. The Program in Palliative Care Education and Practice Questionnaire (German Revised) is psychometrically evaluated for the first time in Iran.

**Methods:**

To measure the psychometric properties, 360 nursing students (BSc, MSc, PhD) and clinical nurses completed the questionnaire. Face and content (CVR and CVI) validity were checked by quantitative and qualitative approach. Construct validity was performed with exploratory and confirmatory factor analysis. The total variance explained was equal to 43%; the internal consistency reported a Cronbach’s alpha of more than 0.7; and the composite reliability was greater than 0.7.

**Results:**

After conducting construct validity and factor analysis, four factors (Knowledge and skill of managing patients’ pain and symptoms, management of ethical and psychological issues in patients, communicating with patients and their families & management of patients’ exposure to grief and attitudes towards death) were extracted. The total variance was equal to (%43) and coefficients of internal consistency were estimated more than 0.7. Also composite reliability was evaluated greater than 0.7.

**Conclusion:**

Persian version of the Program in Palliative Care Education and Practice Questionnaire (German Revised Version; PPCEP-GR) is a valid and reliable questionnaire that can be used to measure the knowledge and performance of nurses and nursing graduates in the field of palliative care.

## Introduction

According to a report by the World Health Organization, about 40 million people need palliative care every year, and 78% of these people live mainly in poor and developing countries. However, only 14% of the people who need this type of care manage to receive it. The global need for palliative care is continuously on the rise as people age in different societies [1]. Palliative care is aimed to improve the quality of life of patients and their families in facing the problems associated with life-threatening diseases. It involves prevention, reduction, early diagnosis, and accurate assessment and treatment of pain and suffering as well as other physical, psychological, social, and spiritual problems [2]. A palliative care approach should be taken into account for people with cancer or any progressive or chronic illness as soon as the diagnosis of a life-threatening illness. Palliative care services should be incorporated at all levels of care in existing health systems [3]. Nurses play a key role in palliative care since among health care professionals, nurses are at the forefront of providing palliative care in almost all health care settings, including inpatient, outpatient, home care, and nursing homes [4].

There is a national and international consensus about the palliative care program, which includes recommendations for familiarizing students of medical sciences with the structure and processes of palliative care and promoting the quality of palliative care. However, evidence suggests that medical graduates have little, if any, knowledge of palliative care. Studies in Germany showed that in this country only 47% of palliative care education institutions have bedside care, and only 59% of them have direct contact with patients in palliative care [5]. The results of Cleary’s study in New York (2020) showed a modest level of knowledge of nurses as far as palliative care and end of life care is concerned [6]. In 2020, Kim measured nurses’ knowledge and attitudes toward palliative care in Mongolia using the 20-item Palliative Care Quiz for Nurses (PCQN). The median PCQN score was 8.0 out of 20, or 39.5% correct, with a range of 3 to 13 [7]. The low self-efficacy of nurses in providing palliative care was reported by Fuoto et al. (2019) [8]. The findings of Iranmanesh et al.‘s (2014) indicated the insufficient knowledge of nurses in the southeast region of Iran regarding palliative care [9]. Several studies have shown that a large number of nurses feel anxious and unprepared to communicate with patients and their families when providing palliative and end-of-life care. According to a study conducted in England, only one out of 9 nurses felt satisfied with providing palliative and end-of-life care, which indicates a deficiency in the training programs of nurses both during education and in practice [10].

There is still no consensus on the measurement tools designed for the evaluation of palliative care training programs [5]. To date, various tools have been used to measure palliative care, including the palliative care quiz for nursing (PCQN) and self-efficacy in palliative care scale (SEPC). However, these tools do not comprehensively measure the different dimensions of the training program and palliative care practice. PCQN is used to measure the theoretical knowledge of nursing care. The items of this tool focus on the philosophy and principles of palliative care, symptom management, and psychological and spiritual care of individuals and families. SEPC is used to evaluate confidence when delivering palliative care. This tool includes three separate subscales (perceived self-efficacy in communication, patient symptom management, and multidisciplinary teamwork). Other notable tools include Frommelt Attitudes Toward Care of the Dying Scale Form B (FATCOD-B) that is used to measure the attitude of doctors and nurses regarding end-of-life care and the comfort/discomfort in caring for the dying, and the Revised Collett–Lester Fear of Death Scale (CL-FODS) used to measure fear of death and death anxiety. None of these tools, however, can be used to measure palliative care education and performance [11].

Therefore, the need for a valid and accurate tool to check the quality and effectiveness of palliative care training programs for nurses is hardly disputable. The Program in Palliative Care Education and Practice (PCEP) was developed by Sullivan and colleagues at Harvard Medical School, Boston, to assess the success of an educational program for physicians and nurses [5]. The program was developed using a multi-step process including panels of experts and peer review, using adult learning theory and Bandura’s concept of self-efficacy. Schulz et al. developed a short version of the questionnaire in German for the evaluation of the undergraduate Palliative Care education (UPCE) program at the University of Witten/Herdke, using items related to the UPCE questionnaire. Later, in 2017, Fetz et al. psychometrically evaluated a short form of this questionnaire in German language to measure the education program and palliative care practices [12]. The Program in Palliative Care Education and Practice Questionnaire (German Revised Version; PCEP-GR henceforth) is a valuable tool to measure nurses’ knowledge of palliative care in four domains of preparation to provide palliative care, attitudes towards palliative care, self-estimation of competence in communication with dying patients and their relatives, and self-estimation of knowledge and skills in palliative care [12, 13]. This questionnaire, in addition to measuring theoretical knowledge, tap into emotional and attitude-related aspects [5]. Therefore, the present research was aimed to perform a psychometric analysis of the Persian version of this questionnaire in Iran.

## Method

This study utilized a cross-sectional methodological design [14].

### Participants

The research population in this study included nursing students as well as staff nurses working in the Intensive Care Unit (ICU) and Oncology departments of teaching hospitals affiliated to Ahvaz Jundishapur University of Medical Sciences in Ahvaz, southwest of Iran. Sampling started on May 10, 2022 and ended on September 11, 2022. The researcher (the first author) visited the ICU and Oncology departments of teaching hospitals and provided the questionnaires to the participants. The participants were eligible to enter the study if they had at least 6 months of clinical work experience (for nurses in ICU) or were a final year bachelor’s student or a student at a higher program in the field of nursing. The participants were selected from the research population through convenience sampling. A total of 360 nurses and nursing students were invited and agreed to participate in the survey. The acceptance rate was 100%.

### Data collection

In order to collect data, a demographic and occupational checklist (age, gender, working or student status, work experience, educational level) as well as PCEP-GR questionnaire, which was designed in form of a 5-point Likert scale (completely disagree to completely agree) were used. Prior to data collection, written informed consent was obtained from the participants, and their information was recorded anonymously. Also, permission to use the PCEP-GR questionnaire was obtained from developers.

### Translation process

At first, the translation of the initial English version of the questionnaire was done in a forward-backward manner by two independent translators fluent in English. The translators were selected in such a way that one of them was fully familiar with the concepts and terms of medical sciences, while the other had no knowledge in this field but had a very good command of English. The two independent Persian translations obtained were examined and revised, and after merging them, a single Persian version of the PCEP-GR questionnaire (PPCEP-GR henceforth) was prepared. Finally, the final Persian version was translated into English and sent to the developer to be compared with the original version of the questionnaire, and it was eventually approved.

### Face validity of the PPCEP-GR

The face validity of the PPCEP-GR was measured by quantitative and qualitative methods. In the quantitative validity analysis, 10 people from the target group (staff nurses and nursing students) were asked to express the importance of the instrument’s items based on a 5-point Likert scale (from completely understandable to not understandable at all). Then, using the quantitative method of item impact, the scores of each item were calculated according to the following formula: Impact Score = Frequency (%) × Importance.

Frequency is the number of people who gave 4 and 5 points to each item, and Importance is the average score obtained from the responses of the participants to the above-mentioned Likert scale. A score higher than 1.5 was considered desirable for each item. The qualitative analysis of face validity was on items that had an impact score of less than 1.5, and it involved face-to-face interviews with the target group on item difficulty and relevance, and ambiguity in understanding the items. In the examination of the face validity of the items that had an impact score of less than 1.5, a face-to-face interview was again conducted with the same target group regarding the examination of the level of difficulty, the degree of appropriateness, and the ambiguity and difficulty in understanding the items [15].

### Content validity

The content validity of the PPCEP-GR was assessed by quantitative and qualitative methods. In the qualitative content validity evaluation phase, PPCEP-GR was given to 10 qualified experts (including 5 nursing professors and 5 clinical nurses who had experience in end-of-life care). They were requested to evaluate the questionnaire after a qualitative review, and provide the necessary feedback based on criteria such as grammar, using appropriate words, placing items in their proper place, and giving appropriate points [16]. In this step, 11 items were corrected in terms of grammar and their position. Also, at this stage, the number of items rose to 38.

Quantitative content validity assessment was based on the opinion of 10 experts, and it involved two indexes of content validity ratio (CVR) and content validity index (CVI). In the CVR index, the necessity of an item was checked. The purpose of the content validity ratio is to select the most important and accurate content. For this purpose, the PPCEP-GR was given to 10 experts, and they were asked to examine and score each item based on a 3-point scale (1. Not necessary; 2. Useful but not necessary; 3. Necessary). Then, if the score obtained by the experts was greater than 0.62 according to the Lawshe table (to determine the minimum value of the index), it would indicate that the relevant item should be included in the questionnaire with a statistical significance level P < 0.05 [15]. The following formula was used to calculate CVR:

1 $$CVR = \frac{{{n_E} - \frac{N}{2}}}{{\frac{N}{2}}}$$

where nE is the number of experts who have considered the item in question as necessary, and N is the total number of experts. CVI is calculated to ensure whether the items are designed in the best way to measure the constructs. For this purpose, in the current research, relevance of items was assessed using a 4-point Likert scale for each of the items (from not relevant to completely relevant) by an expert panel of ten members [17]. After the experts were consulted, Waltz & Bausell index was used to calculate CVI according to the following formula: CVI = Ne/N. Ne is the number of experts who have chosen option 3 and 4, and N is the total number of experts. If the score of an item is more than 0.79, that item remains in the questionnaire. If the CVI score is between 0.70 and 0.79, the item is questionable and needs to be revised, and if the score is less than 0.70, the item is unacceptable and should be removed [18].

### Construct validity

For the purpose of data analysis, we randomly divided the dataset (n = 360) into two subsamples. The first dataset (n = 180) was used to conduct EFA using SPSS version 26, and second the dataset (n = 180) was used to conduct CFA using AMOS version 24. In order to check the construct validity of PPCEP-GR, the first step involved extracting the number of latent factors based on exploratory factor analysis (EFA). To check the adequacy of sampling, the Kaiser-Meyer-Olkin (KMO) test and Bartlett test were performed. A KMO value of more than 0.9 are considered marvelous [19]. Then extraction of latent factors was done based on the maximum likelihood ratio method and Promax rotation. The presence of an item in the factor was determined to be approximately 0.3 based on the following formula: CV = 5.152÷√(n-2) [16]. According to the three indicator rule, there should be at least 3 items for each factor. Communalities with a value less than 0.2 were removed from EFA [17].

In the second step, confirmatory factor analysis (CFA) was performed based on the most common goodness of fit indicators, taking into account the accepted threshold and the maximum likelihood ratio. The assumption of normality was checked based on the skewness index of ± 3 and kurtosis of ± 7. Jaccard and Wan suggested that at least six model fit indices should be presented to show that the model has good fit [20]. The model fit was assessed through a number of fit indices, such as Chi-square (χ2) test, χ2/degree of freedom(df) ratio < 4, goodness-of-fit index (GFI) > 0.90, comparative fit index (CFI) > 0.90, relative Fit Index (RFI) > 0.90, incremental fit index (IFI) > 0.90, and Tucker–Lewis index (TLI) > 0.90, standardized root mean square residual (SRMR) < 0.09, and root mean square error of approximation (RMSEA) < 0.08 [19]. Differences in demographic characteristics between the two groups of participants involved in exploratory factor analysis (EFA) and confirmatory factor analysis (CFA) were tested by Student’s T test for continuous variables and Pearson’s chi-squared test for categorical variables. The limit of statistical significance was set to 5%.

### Reliability

In order to evaluate the internal consistency of PPCEP-GR, Cronbach’s alpha (α), McDonald Omega coefficient (ω), and composite reliability (CR) were estimated at values greater than 0.7, which indicates a good performance [17]. CR was computed only on CFA [19].

## Results

Results showed that the average age of the participants was 31.19 (SD = 7.98). It should be noted that according to Table 1., the demographic characteristics of the participants in the two groups of exploratory (EFA) and confirmatory factor analysis (CFA) were not statistically significant in terms of age, gender, educational attainment, and employment status.

**Table 1:** Sociodemographic characteristics of participants by their inclusion in the CFA or EFA (n = 360)

		CFA	EFA	P
Variable		Mean (± SD)	Mean(± SD)	
Age		34.18 (8.37)	33.78 (6.58)	0.06
		Frequency (%)	Frequency (%)	
Sex	Male	67 (37.2)	79 (43.9)	0.198
	Female	113 (62.80)	101 (56.1)	
Education	Bachelor	159 (88.3)	153 (85.0)	0.096
	MSC	8 (4.5)	18 (10)	
	PhD	13 (7.2)	9 (5)	
Employment status	Clinical	136 (75.5)	140 (77.8)	0.618
	Student	44 (24.5)	40 (22.2)	

Quantitative face validity was evaluated based on item impact method (IIM), and since the calculated impact was higher than 1.5 for each item, face validity was acceptable. The qualitative content validity was also confirmed after the tool was modified by qualified experts, which involved making the necessary grammatical corrections and increasing the number of items to 38. Also, according to the results obtained from the quantitative content validity assessment, three items were removed due to CVR < 0.62 and CVI < 0.70, and according to Lawshe table). Therefore, the number of items at this stage was reduced to 35. KMO was 0.917, and Bartlett’s test was 4158.47 (df = 325, P < 0.001). In the exploratory factor analysis, four factors (i.e., knowledge and skills of managing patients’ pain and symptoms, management of ethical and psychological issues in patients, communication with patients and their relatives, management of patients’ exposure to grief and their attitudes toward death) were extracted. The eigenvalues obtained for these four factors were 3.633, 2.732, 3.172 and 1.563, respectively, and the total variance of the four exploratory factors was 43% (Table 2).

**Table 2:** Factors extracted from PPCEP-GR

Factors	Items	Factor loading	h^2^	λ	% Variance
Knowledge and skill of managing patients’ pain and symptoms	**Q33**: I have sufficient knowledge about the causes of frequent symptoms of patients in need of palliative care.	0.817	0.655	3.633	14.10%
	**Q31**: I have sufficient knowledge of managing shortness of breath or respiratory distress in terminal illness.	0.801	0.601		
	**Q32**: I have sufficient skills to manage shortness of breath or respiratory distress in terminal illness.	0.785	0.619		
	**Q34**: I have sufficient ability to manage frequent symptoms of patients requiring palliative care.	0.785	0.644		
	**Q30**: I have sufficient pain management skills in terminal illness.	0.679	0.557		
	**Q35**: I have sufficient knowledge about the therapeutic effects and adverse effects of painkillers.	0.661	0.443		
Communicating with patients and their families Communicating with patients and their families	**Q25**: I discuss death with patients.	0.911	0.717	3.172	12.20%
	**Q21**: Doctors/nurses have a duty to help patients prepare for death.	0.778	0.597		
	**Q27**: After the death of their patient, I discuss it with the family.	0.669	0.569		
	**Q28**: I answer the patient’s question, “How long will I live?“	0.657	0.454		
	**Q23**: I discuss with patients the possible symptoms of an incurable disease.	0.612	0.555		
	**Q26**: I discuss with the family the imminent death of their patient.	0.572	0.505		
	**Q17**: Upon the request of terminally ill patients, any medication necessary to relieve pain should be given, even if the medication will hasten the patient’s death.	0.375	0.317		
Management of ethical and psychological issues in patients	**Q5**: I am prepared to manage ethical issues arising in the care of patients who are at the end of their life.	0.763	0.533	2.732	10.50%
	**Q3**: I am ready to manage the emotional suffering of patients at the end of their life.	0.740	0.516		
	**Q6**: I am ready to help family members during mourning.	0.644	0.395		
	**Q2**: I am ready to give bad news to the patient about his illness.	0.519	0.383		
	**Q12**: I am prepared to answer the patient’s question “Will I suffer much or be in pain?“	0.518	0.421		
	**Q9**: I am ready to discuss the psychosocial needs and concerns of the patient/family.	0.508	0.289		
	**Q4**: I am ready to discuss with the patient about end-of-life care decisions (such as do-not-resuscitate orders).	0.500	0.300		
	**Q10**: I am ready to deal with cultural differences related to end-of-life care.	0.377	0.430		
Management of patients’ exposure to grief and attitudes towards death	**Q15**: It is impossible to tell patients the truth about the prognosis of the disease and how much hope is left.	0.740	0.537	1.563	6.20%
	**Q19**: Depression in patients with incurable diseases cannot be treated.	0.602	0.430		
	**Q13**: Few measures can be taken to reduce sadness.	0.587	0.303		
	**Q18**: Talking about death discourages terminally ill patients.	0.408	0.230		
	**Q20**: The doctor/nurse is responsible only for the patient. The needs of the family should be taken care of by other professionals.	0.378	0.289		

**Abbreviation: h**^**2**^: Communalities, **λ =** Eigenvalue

**Fig. 1:**
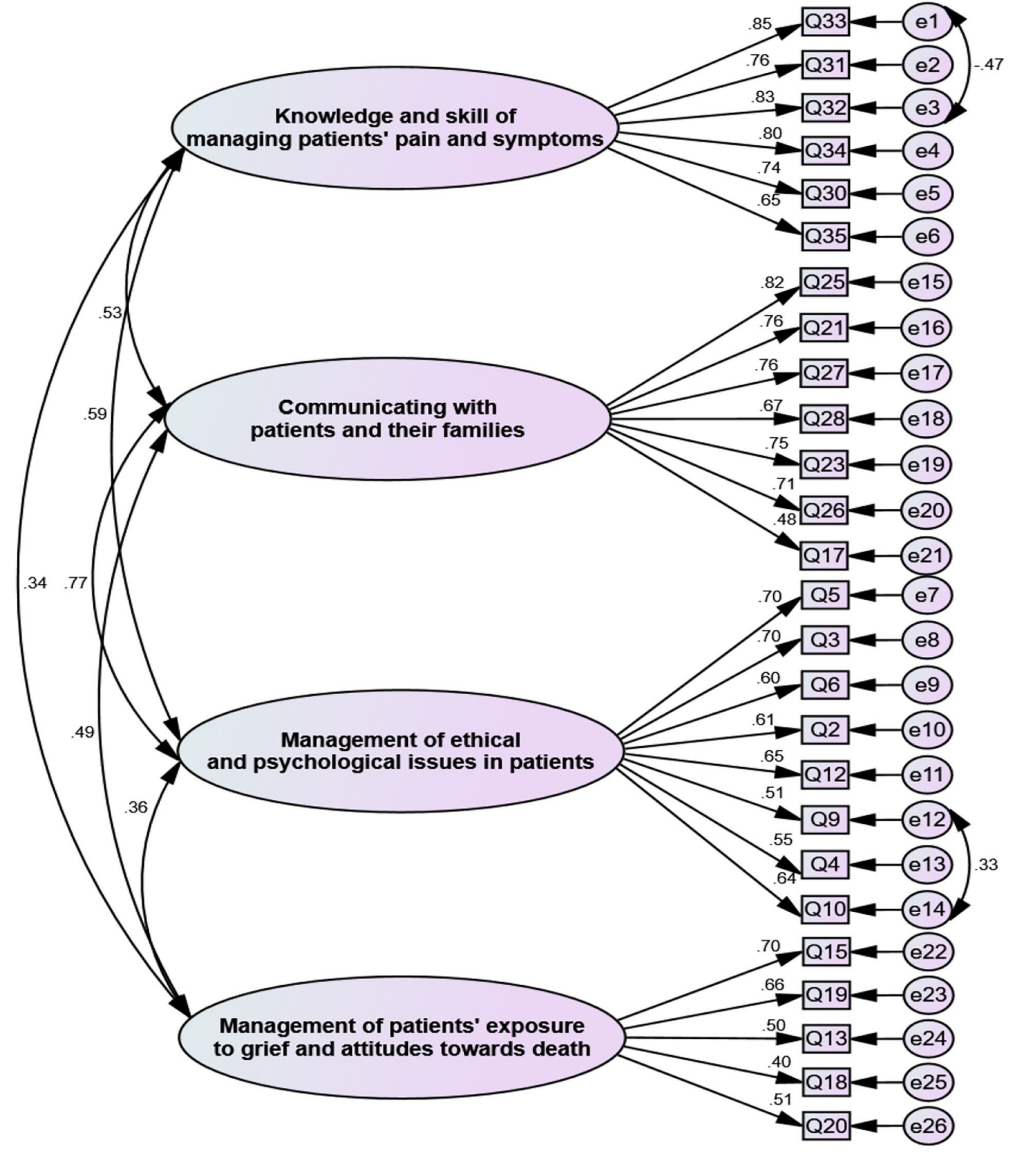
The final structure of PPCEP-GR

In the confirmatory factor analysis, the measured goodness-of-fit indices confirmed the appropriate fit of the final model according to Table 3. According to the final model of the factorial structure of the instrument, the measurement error of Items 1 and 3 (1e to 3e), and Items 12 and 14 (12e to 14e) were correlated (Fig. 1).

**Table 3:** Fit indices of the model according to confirmatory factor analysis

**CMIN/DF**	1.908
**P**	< 0.001
**DF**	291
**CMIN**	555.209
**IFI**	0.934
**TLI**	0.925
**CFI**	0.933
**PNFI**	0.799
**PCFI**	0.835
**RMSEA**	0.050

The internal consistency of the items of the Persian and culturally adapted version of the Program in Palliative Care Education and Practice Questionnaire (German Revised Version; PPCEP-GR) was determined by calculating Cronbach’s alpha for each factor of the instrument separately, and the results showed an acceptable correlation (Table 4).

**Table 4:** Internal consistency for four factors

Factor	α	CR	Ω
Knowledge and skill of managing patients’ pain and symptoms	0.891	0.903	0.893
Management of ethical and psychological issues in patients	0.835	0.798	0.839
Communicating with patients and their families	0.879	0.845	0.873
Management of patients’ exposure to grief and attitudes towards death	0.721	0.702	0.700

**Abbreviation: α**: Cronbach’s alpha, **CR**: Composite reliability, Ω: Omega coefficient

## Discussion

The psychometric analysis of PPCEP-GR was carried out in three steps. The initial instrument included 36 items in 4 domains (preparation to provide palliative care, attitudes towards palliative care, self-estimation of competence in communication with dying patients and their relatives, and self-estimation of knowledge and skills in palliative care). After conducting content validity and construct validity assessments, the number of items of the PPCEP-GR was 26 items in 4 domains (knowledge and skills of managing patients’ pain and symptoms, management of ethical and psychological issues in patients, communication with patients and their relatives, management of patients’ exposure to grief and their attitudes towards death). The changes made to the instrument were in accordance with the participants’ culture, the way nurses provide palliative care, and the educational curriculum of nursing students in Iran.

In the current research, the first factor is knowledge and skills of managing patients’ pain and symptoms. This factor is related to the level of knowledge and skills of nurses and nursing students regarding the management of possible symptoms in patients who need palliative care, which includes pain, side effects of painkillers, shortness of breath, respiratory distress, and other physical symptoms of these patients. In their research, Fetz et al. also considered it necessary to have sufficient competence in controlling common symptoms in patients in need of palliative care, including pain, for providing palliative care. They mentioned it as one of the main dimensions of their tool [5]. Also, in their psychometric analysis of the Spanish version of PCQN, Chover-Sierra et al. dedicated 11 out of the 20 items in the questionnaire to methods of controlling the physical symptoms of the patients, including pain and effective drugs. Also, in their tool, 3 basic domains of palliative care were examined, and management of symptoms and pain was one of these 3 domains [21]. Slåtten et al. tested and validated the nurses’ core competence in palliative care (NCPC) instrument which is used to measure the competence of nurses in providing palliative care. One of the 5 basic domains of NCPC is the knowledge of symptom management including pain, nausea, anxiety, fatigue and other physical symptoms, which is in line with the present study [22].

The second factor in the current research is the management of ethical and psychological issues in patients who need palliative care. This domain examines the psychological issues as well as the ethical considerations that arise when providing palliative care. Arahata et al. developed a tool to measure the knowledge and attitude of nurses in palliative care. Their tool included 10 domains, and one of these domains directly measures ethical issues and considerations in end-of-life care and palliative care [23]. Nakazawa et al., in their assessment of the Palliative Care Knowledge Test (PCKT), which measures nurses’ palliative care knowledge using 20 items in 5 basic domains, mentioned psychological problems and issues as an essential part of nurses’ palliative care knowledge [24].

The third factor in the current research was the ability to communicate with patients and their families. In a review study, Soikkeli-Jalonen et al. examined the available tools used for measuring the knowledge and skills related to palliative care in intensive nursing care. According to their results, establishing correct and effective communication with patients and their relatives in order to involve both of them in making treatment decisions and providing reliable and dependable information was one of the basic domains of any tool used for measuring knowledge of palliative care in nurses [25]. According to Fetz et al., effective communication with patients and their family is emphasized in the Palliative Care Training and Practice Program of Harvard University. Also, in the development and design of their tool, they proposed 4 basic domains, with competency in communication with dying patients and their relatives being one of the important domains [5].

The fourth factor is the management of patients’ exposure to grief and their attitude towards death. Iranmanesh et al. highlighted the need to pay attention to the importance of attitudes towards death in different societies, beliefs and cultures when measuring the level of knowledge of nurses in relation to palliative care [9]. According to Witkamp et al., attitude and perspective of nurses are important components in providing palliative care. In the tool they used to measure the knowledge and perspective of nurses in relation to palliative care, the nurses’ attitude towards providing this care was one of the important factors in this type of care [26]. Soikkeli-Jalonen et al. stressed the importance of having the right attitude in making the right care decisions and providing appropriate palliative care for patients in nurses as one of the prominent and important points in most palliative care knowledge measurement tools [25].

Since PCEP has been previously evaluated psychometrically only in Germany, we compared the statistical results of the current study with those of the German version. The KMO value obtained for the German version is 0.81, which is acceptable and consistent with the KMO value we calculated in the present study (0.917). Also, the percentage of total variance of the German version was 40.20%, and that in the present study was 43% which was explained by the four factors. Finally, the Cronbach’s alpha coefficients in the 4 domains of the German questionnaire were 0.83, 0.82, 0.75 and 0.66, and those in the present study were 0.89, 0.83, 0.87, and 0.72; this comparison assessed how the internal consistency reported to be consistent ​​ [5].

## Conclusion

In the present study, a valid and reliable tool used for measuring the knowledge of nurses was psychometrically assessed, and an attempt was made to provide sufficient information about the process of evaluating the validity and reliability of this tool. Also, the results of the study showed that the questionnaire has desirable psychometric properties and the necessary rigor and validity to measure the knowledge and performance of palliative care in nursing graduates. However, some more research on Persian version considering aspects of validity and reliability we were not able to investigate is necessary. Other limitation of our study was that the psychometric analysis of this tool only involved the population of clinical nurses and nursing students of teaching hospitals affiliated to Ahvaz University of Medical Sciences. Therefore, it is recommended that the psychometric properties of this tool be evaluated in other populations and groups that have palliative care experience. Another limitation of our study is that it is difficult to objectively measure knowledge and practical aspects of providing palliative care and communicating with patients and relatives using this tool. Also, in order to complement any evaluation based on the psychometrically analyzed questionnaire adapted to the context of Iran, the use of objective structured clinical examinations (OSCE) is recommended to obtain more valuable and solid behavioral outcome data. The last limitation of this study was that we used convenience sampling method, which may affect the generalizability of the results. Further study is required to verify the psychometric properties of the Persian version of PPCEP in a larger sample. Also future work needs to be done on other professionals, especially physicians, who are also important team members of palliative and hospice care.

## Data Availability

The datasets used and analyzed during the current study are available from the corresponding author [SM] on reasonable request.

## Abbreviations

PCEP-GR: Program in Palliative Care Education and Practice Questionnaire (German Revised)
PPCEP-GR: Persian version of the Program in Palliative Care Education and Practice Questionnaire (German Revised)
CMIN/DF: Minimum Discrepancy Function by Degrees of Freedom divided
P: (Chi-squared)- p-value
DF: Degrees of Freedom
IFI (Delta2): Incremental fit index
TLI (rho2): Tucker-Lewis Index
CFI: Comparative of Fit Index
PNFI: Parsimonious Normed Fit Index
PCFI: Parsimonious Comparative Fit Index
RMSEA: Root Mean Square Error of Approximation

## References

[1] WH. O: Palliative Care: World Health Organization; [Available from: https://www.who.int/news-room/fact-sheets/detail/palliative-care. 2020.

[2] Getie A, Wondmieneh A, Bimerew M, Gedefaw G, Demis A (2021). Knowledge on Palliative Care and Associated factors among nurses in Ethiopia: a systematic review and Meta-analysis. Pain Res Manage.

[3] Murray SA, Kendall M, Mitchell G, Moine S, Amblàs-Novellas J, Boyd K (2017). Palliative care from diagnosis to death. BMJ (Clinical research ed).

[4] Sekse RJT, Hunskår I, Ellingsen S (2018). The nurse’s role in palliative care: a qualitative meta-synthesis. J Clin Nurs.

[5] Fetz K, Wenzel-Meyburg U, Schulz-Quach C (2017). Validation of the german revised version of the program in palliative care education and practice questionnaire (PCEP-GR). BMC Palliat care.

[6] Cleary AS (2020). Graduating nurses’ knowledge of palliative and end-of-life care. Int J Palliat Nurs.

[7] Kim JS, Kim J, Gelegjamts D (2020). Knowledge, attitude and self-efficacy towards palliative care among nurses in Mongolia: a cross-sectional descriptive study. PLoS ONE.

[8] Fuoto A, Turner KM (2019). Palliative care nursing communication: an evaluation of the COMFORT model. J hospice Palliat nursing: JHPN : official J Hospice Palliat Nurses Association.

[9] Iranmanesh S, Razban F, Tirgari B, Zahra G (2014). Nurses’ knowledge about palliative care in Southeast Iran. Palliat Support Care.

[10] Glover TL, Garvan C, Nealis RM, Citty SW, Derrico DJ (2017). Improving end-of-life care knowledge among senior baccalaureate nursing students. Am J Hosp palliat Care.

[11] Frey RA, Gott M, Neil H (2013). Instruments used to measure the effectiveness of palliative care education initiatives at the undergraduate level: a critical literature review. BMJ supportive & palliative care.

[12] Fetz K. Research Methodology in Palliative Care – an evidence-based Approach to improve Educational and Clinical Assessment. Witten/ Herdecke university; 2020.

[13] Fetz K. Assessments in der Palliativausbildung und –versorgung. In M. W. Schnell, C. Schulz-Quach, & C. Dunger, Herausgeber, Assessments in der Palliativausbildung und -versorgung: Eine psychometrische Instrumententestung (S. 21–102). Springer Fachmedien. 10.1007/978-3-658-35965-2_3. 2022 a.

[14] Polit FD, Beck TC. Essentials of nursing research: appraising evidence for nursing practice, Ninth edition edn. Philadelphia: Wolters Kluwer Health; 2018.

[15] Sharifnia H, Zareiyan A, Ebadi A. Test Development process in Health Sciences. 3th ed. jph; 2022.

[16] Sharif Nia H, Hosseini L, Ashghali Farahani M, Froelicher ES (2023). Development and validation of care stress management scale in family caregivers for people with Alzheimer: a sequential-exploratory mixed-method study. BMC Geriatr.

[17] Sharif Nia H, Kaur H, Fomani FK, Rahmatpour P, Kaveh O, Pahlevan Sharif S, Venugopal AV, Hosseini L (2021). Psychometric Properties of the impact of events scale-revised (IES-R) among General Iranian Population during the COVID-19 pandemic. Front Psychiatry.

[18] Rahmatpour P, Peyrovi H, Sharif Nia H (2021). Development and psychometric evaluation of postgraduate nursing student academic satisfaction scale. Nurs Open.

[19] Hosseini L, Sharif Nia H, Ashghali Farahani M (2022). Development and psychometric evaluation of Family Caregivers’ hardiness scale: a sequential-exploratory mixed-method study. Front Psychol.

[20] Jaccard J, Wan C. LISREL Approaches to Interaction Effects in Multiple Regression. In. Thousand Oaks, California; 1996.

[21] Chover-Sierra E, Martínez-Sabater A, Lapeña-Moñux YR (2017). Correction: an instrument to measure nurses’ knowledge in palliative care: validation of the spanish version of Palliative Care Quiz for Nurses. PLoS ONE.

[22] Slåtten K, Hatlevik O, Fagerström L. Validation of a new instrument for self-assessment of nurses’ core competencies in palliative care. Nurs Res Pract 2014, 2014:615498. 10.1155/2014/615498 PMC4124716 25132989

[23] Arahata T, Miyashita M, Takenouchi S, Tamura K, Kizawa Y (2018). Development of an instrument for evaluating nurses’ knowledge and attitude toward end-of-Life Care: end-of-life nursing Education Consortium-Japan Core Quiz. J hospice Palliat nursing: JHPN : official J Hospice Palliat Nurses Association.

[24] Nakazawa Y, Miyashita M, Morita T, Umeda M, Oyagi Y, Ogasawara T (2009). The palliative care knowledge test: reliability and validity of an instrument to measure palliative care knowledge among health professionals. Palliat Med.

[25] Soikkeli-jalonen A, Stolt M, Hupli M, Lemetti T (2019). Instrument for assessing nurses palliative care knowledge and skills in specialised care setting: an integrative review. J Clin Nurs.

[26] Witkamp FE, van Zuylen L, van der Rijt CC, van der Heide A (2013). Validation of the Rotterdam MOVE2PC Questionnaire for assessment of nurses’ knowledge and opinions on palliative care. Res Nurs Health.

